# Correlation between histological invasiveness and the computed tomography value in pure ground-glass nodules

**DOI:** 10.1007/s00595-015-1208-1

**Published:** 2015-06-30

**Authors:** Akihiko Kitami, Fumitoshi Sano, Shoko Hayashi, Kosuke Suzuki, Shugo Uematsu, Yoshito Kamio, Takashi Suzuki, Mitsutaka Kadokura, Mutsuko Omatsu, Toshiaki Kunimura

**Affiliations:** Respiratory Disease Center, Showa University Northern Yokohama Hospital, 35-1 Chigasaki-Chuo, Tsuzuki-ku, Yokohama-City, 224-8503 Japan; Department of Clinical Diagnostic Pathology, Showa University Northern Yokohama Hospital, Yokohama, Japan; Division of Chest Surgery, Showa University School of Medicine, Tokyo, Japan

**Keywords:** Lung cancer, Diagnosis, Surgery, Computed tomography

## Abstract

**Purpose:**

The purpose of this study was to evaluate the correlation between histological invasiveness and the computed tomography (CT) value and size in pure ground-glass nodules (GGNs) to determine optimal “follow-up or resection” strategies.

**Methods:**

Between 2001 and 2014, 78 resected, pure GGNs were retrospectively evaluated. The maximum diameter and CT value of pure GGNs were measured using a computer graphics support system.

**Results:**

All GGNs with a maximum diameter ≤10 mm and CT value ≤−600 Hounsfield units (HU) were considered to be noninvasive lesions, while 21 of 26 (81 %) with a maximum diameter >10 mm and CT value >−600 HU were considered to be invasive lesions. With respect to the correlation between each histological type and pure GGN with a maximum diameter ≤10 mm and CT value ≤−600 HU, the specificity was 90 % and the sensitivity and negative predictive value were both 100 % in atypical adenomatous hyperplasia (AAH), while the specificity was 58 % and the sensitivity and positive predictive value were 0 % in minimally invasive and invasive adenocarcinoma.

**Conclusion:**

Pure GGNs with a maximum diameter of ≤10 mm and CT value of ≤−600 HU are nearly always pre-invasive lesions; therefore, surgery should be carefully selected in such patients.

## Introduction

A ground-glass opacity (GGO) is defined as a shadow completely occupied by a hazy increased attenuation of the lung, with reservation of the bronchial and vascular margins in the lesion on HRCT. Among nodules detected on computed tomography (CT), a substantial number have GGO and are therefore referred to as ground-glass nodules (GGNs). Nonsolid GGNs (pure GGNs) are homogeneous lesions; however, they exhibit variations in the CT findings with respect to value and size. The histological features of persistent pure GGNs include atypical adenomatous hyperplasia (AAH), adenocarcinoma in situ (AIS), minimally invasive adenocarcinoma (MIA), and occasionally invasive adenocarcinoma (Ad.) such as lepidic predominant adenocarcinoma (LPA). The purpose of this study was to evaluate the correlation between histological invasiveness and the CT value and size in pure GGNs to determine optimal “follow-up or resection” strategies for lesions displaying pure GGNs.

## Patients and methods

We recommended resection of GGN lesions that did not decrease in size during 3 months of follow-up prior to 2009. Since 2010, we defined GGN lesions with a maximum diameter >10 mm and CT value >−600 HU [1].

Between April 2001 and September 2014, a total of 84 pure GGNs in 81 patients were resected at our hospital. Of these, 78 nodules in 72 patients with pure GGNs ≤30 mm in size were evaluated in this retrospective analysis. Five patients had multiple pure GGNs, and three patients had pure GGNs and solid or partially solid adenocarcinomas.

During this period, 45 pure GGNs in 39 patients were followed up using HRCT at 4–6 months interval. Forty-one GGNs were lesions with a maximum diameter ≤10 mm or CT value ≤−600 HU, and four GGNs were lesions with a maximum diameter >10 mm and CT value >−600 HU.

**Fig. 1:**
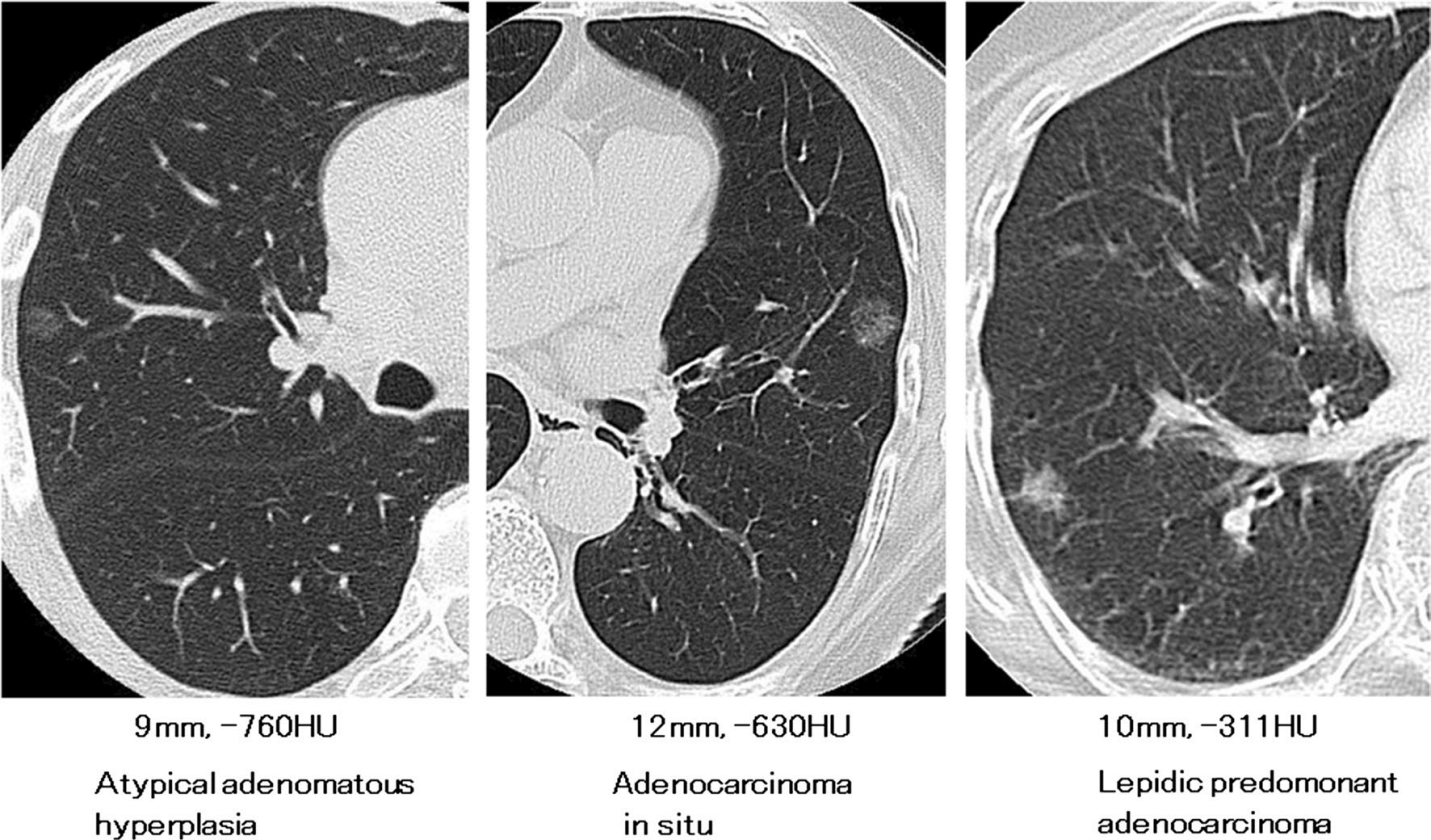
Radiologic findings of pure GGN

Non-enhanced CT scans were performed from lung apices to bases during breath-holding at mid-inspiration using a CT scanner (Aquilion 64, Toshiba, Tokyo, Japan). The helical scanning protocol was as follows: 120 kVp; 200 mA; 0.5-s scanning time; window level, −500 Hounsfield units (HU); window width, 1600 HU; and a 512 × 512 matrix corresponding to a pixel size of approximately 0.6 mm. HRCT images were reconstructed at 0.5-mm intervals with a high-spiral-frequency algorithm (bone algorithm). Pure GGN was defined as a shadow completely occupied by a hazy increased attenuation of the lung, with reservation of the bronchial and vascular margins in the lesion with no solid regions on HRCT. The maximum diameter and CT value were measured using a computer graphics support system (HOPE/DrABLE-EX. Fujitsu, Tokyo, Japan). The CT value of each GGN lesion was measured in two or three areas excluding portions of apparent vessels, and the highest value in nonhomogeneous lesions was selected. Histological diagnoses were based on the new classification of lung adenocarcinoma proposed by the International Association for the Study of Lung Cancer, the American Thoracic Society, and the European Respiratory Society and classified as adenocarcinoma in situ (AIS), minimally invasive adenocarcinoma (MIA), and invasive adenocarcinoma (Ad.) [lepidic predominant adenocarcinoma (LPA) and other histological subtypes] [2]. We classified AAH and AIS as pre-invasive lesions, and MIA and Ad. as invasive lesions. Radiologic findings of three representative cases are shown in Fig. 1.

Statistical analyses were performed using SPSS II (SPSS Inc; Chicago, IL, USA). All data were expressed as the mean ± standard deviation (SD). The differences in mean and SD values were analyzed between groups using a two-tailed Student’s *t* test. Between-group differences with *p* values less than 0.05 were considered to be statistically significant.

## Results

Patients ranged in age from 39 to 83 years (median, 64 years). Forty patients were female and 32 were male. The maximum tumor dimension was ≤10 mm in 30 nodules, 11–20 mm in 36 nodules, and 21–30 mm in 11 nodules. The mean maximum tumor dimension of all cases was 12.9 ± 6.1 mm. The mean CT value of all cases was −569 ± 126 HU.

**Fig. 2:**
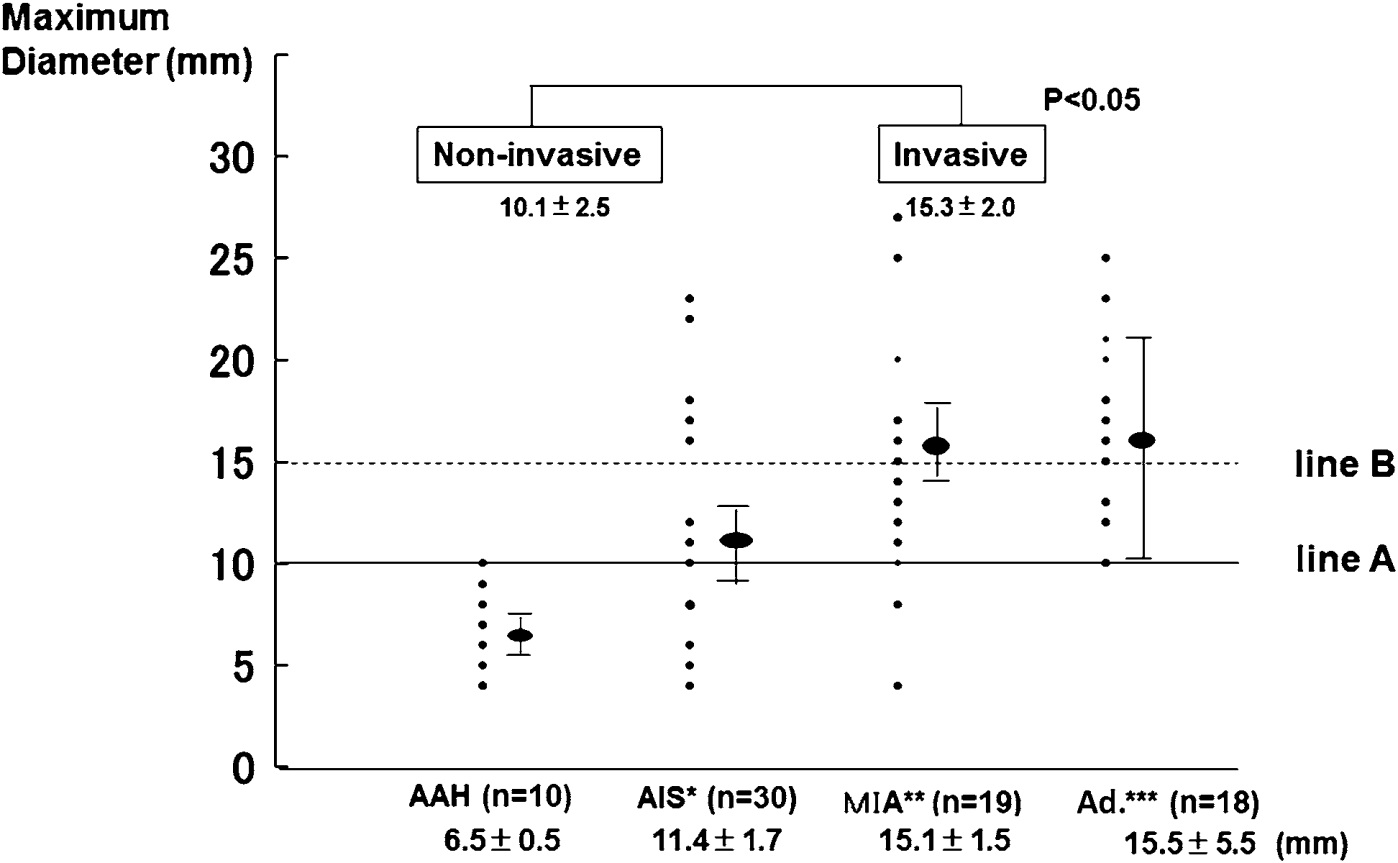
The plots of the maximum diameter in AAH, AIS, MIA, and Ad. The percentage of invasive lesions was 19 % (6/31) for lesions ≤10 mm (*line A*), while it was 38 % (20/53) for lesions ≤15 mm (*line B*). The difference between the noninvasive and invasive lesions was significant (*p* < 0.05). Some plots with the same size overlap at the same point. *Bars* mean ± SD. AIS* adenocarcinoma in situ, MIA** minimally invasive adenocarcinoma, Ad.*** invasive adenocarcinoma

**Fig. 3:**
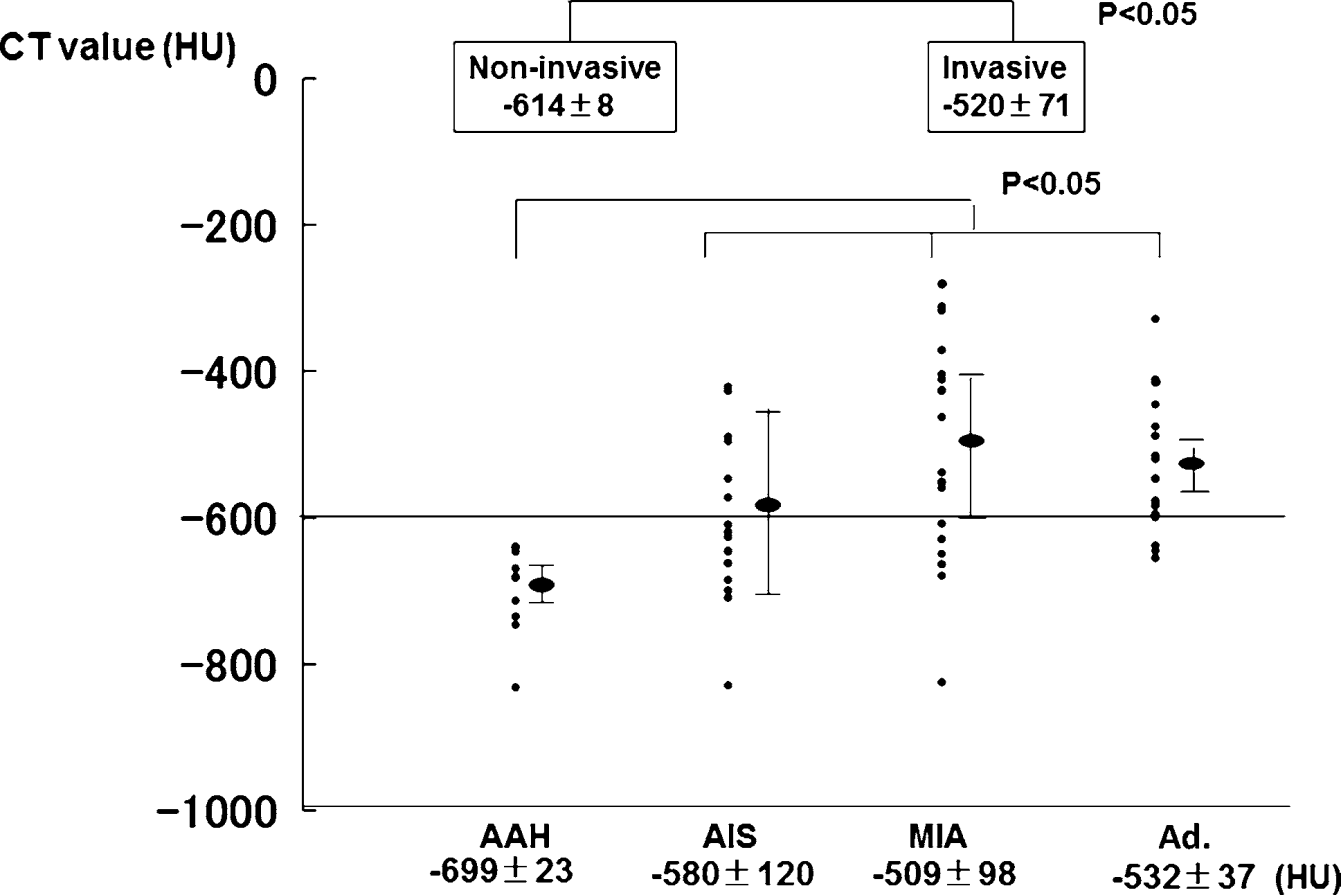
CT values in AAH, AIS, MIA, and Ad. All AAH lesions were ≤−600 HU and had significantly lower CT values than AIS, MIA, and Ad. The differences between AIS and MIA and between the noninvasive and invasive lesions were significant (*p* < 0.05). No significant difference in the CT values was observed between MIA and Ad. Some plots with the same CT value overlap at the same point. *Bars* mean ± SD

**Fig. 4:**
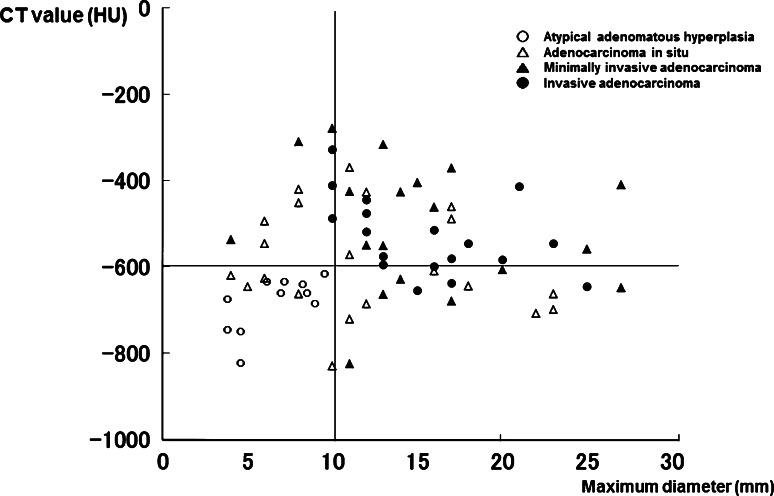
Relationship between the CT value and maximum diameter of pure GGN lesions. All GGN lesions with a maximum diameter ≤10 mm and CT value ≤−600 HU were noninvasive lesions, while 21 of 26 (81 %) GGN lesions with a maximum diameter >10 mm and CT value >−600 HU were invasive lesions. Some plots with the same size and CT value overlap at the same point

The mean interval from initial detection of focal GGO to pulmonary resection was 18 months (range 3–130 months). Pathologic specimens demonstrated AAH in 10 nodules, AIS in 30 nodules, MIA in 19 nodules, and Ad. in 18 nodules, including 14 cases of LPA and lymphoproliferative disorder in 1 nodule. Surgical procedures included lobectomy in 30 nodules, segmentectomy in 15 nodules, and partial resection in 32 nodules. None of the 47 patients, except for 1 with solid adenocarcinoma who underwent lymph node dissection and sampling, had lymph node involvement. All patients, except for two who died from other diseases and three who experienced recurrence accompanied with solid adenocarcinoma, have survived with no evidence of tumor recurrence to date, at a median follow-up of 46 months. Figure 2 shows the distribution of the maximum diameter in AAH, AIS, MIA, and Ad. The mean maximum diameter in AAH, AIS, MIA, and Ad was 6.5 ± 0.5 mm, 11.4 ± 1.7 mm, 15.1 ± 1.5 mm, and 15.1 ± 5.5 mm, respectively. The percentage of invasive lesions was 19 % (6/31) among lesions ≤10 mm (line A) and 38 % (20/53) in lesions ≤15 mm (line B). The difference between the noninvasive and invasive lesions was statistically significant (*p* < 0.05). Figure 3 shows the distribution of the CT values in AAH, AIS, MIA, and Ad. The mean CT value in AAH, AIS, MIA, and Ad was −699 ± 23 HU, −580 ± 120 HU, −509 ± 98 HU, and −532 ± 37 HU. All AAH lesions were less than −600 HU and had significantly lower CT values than AIS, MIA, and Ad. The differences between AIS and MIA or between the noninvasive and invasive lesions were statistically significant (*p* < 0.05). No significant difference in the CT values was observed between MIA and Ad. Figure 4 illustrates the relationship between the CT value and maximum diameter of pure GGN lesions. All GGN lesions with a maximum diameter ≤10 mm and CT value ≤−600 HU were considered to be noninvasive lesions, while 21 of 26 (81 %) with a maximum diameter >10 mm and CT value >−600 HU were considered to be invasive lesions. With respect to the correlation between each histological type and pure GGN lesions with a maximum diameter ≤10 mm and CT value ≤−600 HU, the specificity was 90 % and the sensitivity and negative predictive values were 100 % in AAH, while the specificity was 58 %, and the sensitivity and positive predictive values were 0 % in MIA and Ad (Table 1).

**Table 1:** Correlation of histology and pure GGN lesions with a maximum diameter ≤10 mm and CT value ≤−600 HU

AAH	AAH	nonAAH	Total		
≤−600 HU and ≤10 mm	10	7	17	Specificity	90 %
>−600 HU or > 10 mm	0	60	60	Sensitivity	100 %
Total	10	67	77	Positive predictive value	59 %
				Negative predictive value	100 %

AIS	AIS	non AIS	Total		
≤−600 HU and ≤10 mm	7	10	17	Specificity	76 %
>−600 HU or >10 mm	23	28	60	Sensitivity	23 %
Total	30	37	77	Positive predictive value	41 %
				Negative predictive value	47 %

MIA + Ad.	MIA + Ad.	Others	Total		
≤−600 HU and ≤10 mm	0	17	17	Specificity	58 %
>−600 HU or >10 mm	37	23	60	Sensitivity	0 %
Total	37	40	77	Positive predictive value	0 %
				Negative predictive value	38 %

As concerns AAH, specificity is 90 %, sensitivity and negative predictive value are 100 %. While, in MIA and invasive adenocarcinoma, specificity is 58 %, sensitivity and positive predictive value are 0 %

In 45 follow-up cases of GGNs, two (50 %) of four lesions with a maximum diameter >10 mm and CT value >−600 HU and 11 (24 %) of 41 lesions ≤10 mm or CT value ≤−600 HU had findings of increased size or CT number. The median follow-up period was 51 months.

## Discussion

Many radiologic studies of small lung adenocarcinomas have demonstrated a strong correlation between the CT findings and pathologic features [3, 4]. Several groups have classified small lung lesions into the nonsolid (pure) GGO type, partly solid (mixed) GGO type, and solid type and have suggested that the pure type and mixed type with small solid components are nearly always noninvasive carcinomas [5–8]. However, it is occasionally difficult to differentiate between pure and mixed GGO and between high-density GGO and a solid tumor. Suzuki et al. classified homogeneous (so-called “pure”) GGO into pure GGO and semiconsolidation to evaluate the differences in the density within the tumor and determined that, pathologically, semiconsolidation tends to be adenocarcinoma with invasive foci [9]. Although the differentiation between pure GGN and semiconsolidation was unclear in their report, the density within homogeneous GGNs appears to be an important factor for predicting tumor invasiveness. We speculated that pure GGNs should be defined as homogeneous hazy lesions, for which CT values were ≤−300 HU in the present analysis. Because −300 HU is the limitation of differentiation between vessels, we classified lesions with CT values >−300 HU to be solid nodules.

Several groups have used quantitative densitometric methodologies to evaluate GGN lesions [10–13]. Ikeda et al. reported that the 75th percentile CT value analyzed using three-dimensional computerized quantification of the GGO lesions was the optimal value for differentiating between AAH, BAC, and adenocarcinoma. These investigators noted that a CT cutoff value of −584 HU was optimal for differentiating AAH and BAC, and −472 HU was optimal for differentiating BAC and adenocarcinoma [11]. Nomori et al. used histograms of CT pixel numbers for AAH and nonmucinous adenocarcinoma to quantify the peaks and mean numbers of CT pixels and found the mean CT values to be −697 ± 56 HU for AAH lesions and −541 ± 73 HU for BAC lesions. These investigators noted that the peak CT value on the histogram was the most frequent value observed in the tumor and that the effect of vessels and bronchi within the tumor can be ignored [12]. The CT value of each GGN lesion was measured in two or three areas, excluding the apparent vessels, in this study, and was the selected highest value in nonhomogeneous lesions. The present study demonstrates that a CT value of −600 HU may represent a cutoff value demarcating AAH versus invasive lesions. We believe that this finding could significantly impact the selection of therapeutic strategies for GGN lesions.

Whether GGN lesions that are suspected to be pre-invasive or minimally invasive adenocarcinoma should be resected or followed up also remains controversial. Recently, several groups have reported that for pre-invasive lesions identified on HRCT, careful observation without surgical intervention represents a valid treatment option [14–16]. According to the interim guidelines suggested by Godoy and Nadich, isolated lesions with pure GGNs that are ≤5 mm do not necessarily require CT follow-up, since they nearly always represent foci of AAH; however, for lesions between 5 and 10 mm, follow-up is mandatory pending better definition of their true nature. Lesions >1 cm should be assumed to be so-called BAC or invasive adenocarcinoma; however surgery should be considered, particularly if the nodule is growing or if an increase in attenuation or development of a solid component is observed [17].

Diligent long-term follow-up of the natural history of pure GGN and should be conducted to determine whether surgical intervention is acceptable or unnecessary [18]. However, we believe that early detection and early therapy of some primary lung cancers with pure GGNs are important for improving individual prognoses for the following reasons. First, pure GGN lesions are not all histologically pre-invasive, as some, such as MIA and LPA, have an Ad. component. Second, several groups have reported the concept of a multi-step progression from AAH through localized BAC (AIS and MIA) to advanced adenocarcinoma with a replacement growth pattern (LPA and other type of Ad.) [19–21]. Third, patients with LPA have a lower 5-year survival rate (approximately 93 %) than patients with AIS and MIA (100 %) [22–25]. Our therapeutic strategy for pure GGNs is to avoid unnecessary surgery for AAH and therapeutic delays for Ad.

There are some limitations associated with this study, such as a retrospective study design and small number of surgical cases. However, we revealed a correlation between the histology and pure GGN lesions with a maximum diameter ≤10 mm and CT value ≤−600 HU. With respect to AAH, the specificity was 90 % and the sensitivity and negative predictive values were both 100 %. In contrast to MIA and Ad., the specificity was 58 % and the sensitivity and positive predictive values were both 0 %. According to these results, we propose the follow-up criteria of a maximum diameter ≤10 mm and CT value ≤−600 HU for pure GGNs, and unnecessary surgery for AAH and therapeutic delay for Ad. can be avoided.

## Conclusion

Pure GGNs at a maximum diameter of ≤10 mm and CT value of ≤−600 HU are nearly always pre-invasive lesions; therefore, surgery should be carefully selected in such patients.
